# Effect of supplementation of butylated hydroxytoluene on post-thaw sperm viability, motility and membrane integrity of Hariana bulls

**DOI:** 10.14202/vetworld.2015.808-812

**Published:** 2015-06-30

**Authors:** Akhil Patel, Atul Saxena, Dilip Kumar Swain, Dushyant Yadav, Sanjay Singh Yadav, Abhishek Kumar, Anuj Kumar

**Affiliations:** 1 Department of Veterinary Obstetrics & Gynaecology, College of Veterinary Science & Animal Husbandry, U.P. Pandit Deendayal Upadhayaya Pashu Chikitsa Vigyan Vishwavidyalaya Evam Go Anusandhan Sansthan, Mathura - 281 001, Uttar Pradesh, India; 2 Department of Veterinary Physiology, College of Veterinary Science & Animal Husbandry, U.P. Pandit Deendayal Upadhayaya Pashu Chikitsa Vigyan Vishwavidyalaya Evam Go Anusandhan Sansthan, Mathura - 281 001, Uttar Pradesh, India

**Keywords:** sperm, viability, butylated hydroxytoluene, membrane integrity, antioxidant, Hariana bull

## Abstract

**Aim:**

This study was aimed to see the beneficial effect of butylated hydroxytoluene (BHT) as a semen additive of Hariana bull semen.

**Materials and Methods:**

The study was carried out in Hariana bulls. Twenty-four ejaculates from two bulls were used for this study. Each ejaculate was extended with standard glycerolated egg yolk tris extender and supplemented with BHT at two concentrations as 0.5 mM (T1) and 1.0 mM (T2). After dilution, equilibration and 24 h of cryopreservation, the samples were analyzed for progressive motility, sperm viability and membrane integrity.

**Results:**

Progressive motility, sperm viability and sperm membrane integrity were significantly (p<0.05) increased in the samples fortified with BHT as compared to the control during the process of cryopreservation and thawing. The BHT concentration of 1 mM revealed better results as compared to 0.5 mM.

**Conclusion:**

Addition of 1.0 mM BHT was found better in cryopreservation of Hariana bull semen compared to 0.5 mM BHT and control samples. The addition of BHT has improved the sperm quality by acting as an antioxidant thereby reducing the lipid peroxidation of the sperms.

## Introduction

Cryopreservation of sperm is the integral part of a successful artificial insemination program, which not only depends on the prefreeze sperm features, but also by the freezing protocols [1]. Cryopreservation ensures long-term storage of sperm and its subsequent use in the breeding of domestic as well as wild animals. In spite of lots of development in cryopreservation and cryopreservation protocols, the sperms suffer from irreversible damage both at structural and functional level leading to a reduction in viability, motility, and fertility [2].

Free radical generation is a major limitation during the process of sperm cryopreservation, which is generated from the non-viable sperms as well as from the extenders, which contain the molecular oxygen [3]. The free radicals target the sperm plasma membrane, and in specific the polyunsaturated fatty acids, membrane proteins, acrosome and sperm DNA causing lipid peroxidation of membrane lipids, chromatin cross-linking and fragmentation of sperm DNA [4]. These changes induce irreversible cell damage and sperms undergo induced apoptosis causing a consequent reduction in the sperm quality. Generation of free radicals during cryopreservation cannot be inhibited, but can be reduced by the introduction of suitable antioxidants in the extenders used for sperm cryopreservation [5,6]. In the recent past, many of the laboratories are focusing on various antioxidants and their dose dependent incorporation in the semen extenders to reduce the generation of free radicals and their deleterious effect on sperm [6-8].

Butylated hydroxytoluene (BHT) is a phenolic antioxidant being supplemented in the semen extender as a measure to prevent the membrane permeability changes in the sperms during cryopreservation [9]. BHT also exhibits antiviral activity and has been associated with the inactivation of lipid-containing viruses [10]. Chemically BHT is a synthetic analog of Vitamin E, and is involved in the auto-oxidation reaction, thereby converting the peroxy radicals to hydroxyperoxides [11]. These may be the best possible reasons for which, BHT has been used as a potential antioxidant additive in a number of species, but no literature is available in the Hariana bulls till date.

Antioxidant addition has been emerged as one of the most powerful way to overcome the excessive generation of free radicals during the time of semen cryopreservation and hence most of the laboratories are involved extending the semen with antioxidants. We have already studied the beneficial effects of Vitamin E, Vitamin C and various proteins which provide cryo-protection to the sperms during freeze-thaw process (data unpublished), however, literature is scanty regarding the incorporation of BHT in semen extender, as an antioxidant to protect sperm cells during cryopreservation. This provided us an open area to design our study to observe the effect of BHT addition in the semen extenders for its antioxidant effect reflecting protection to sperm cells.

## Materials and Methods

### Ethical approval

No ethical permission was required to conduct the study as the method of semen collection was noninvasive. However semen collection was done as per standard collection method without harming animal.

### Experimental animals

The present study was conducted on Hariana bulls of the age group between 5.5 and 6.5 years and weighing between 450 and 500 kg body weight, reared at the University Instructional Livestock Farm Complex, College of Veterinary Sciences, U.P. Pandit Deen Dayal Upadhayaya Pashu Chikitsa Vigyan Vishwavidyalaya Evam Go Anusandhan Sansthan (DUVASU), Mathura which is situated in a semiarid zone of Northern part of India, in the state of Uttar Pradesh. The bulls were fed 21.15 kg of greens, 4.83 kg of concentrate including service ration and 4.95-5.77 kg of wheat straw per bull per day. Bulls were apparently free of infection and were undergone for regular vaccination as per the prescribed manual of certified semen laboratory.

### Semen collection

A biweekly semen collection schedule was followed during the entire period of the study, which was carried out during January to March of year 2014. Semen was collected directly into a clean dry graduated centrifuge tube attached to the latex cone of the AV. Immediately after collection, tube containing semen were marked, transferred to the laboratory and placed in the water bath at 32-34°C for physico-morphological studies.

### Evaluation of seminal attributes

The collected semen was evaluated (as per the standard guidelines framed in the semen biology laboratory, DUVASU, Mathura) and those which fulfill the criteria for cryopreservation were extended with glycerolated egg yolk tris (GEYT) extender with two different concentrations (0.5 and 1.0 mM) of BHT. Three stages of sperm evaluation were carried out *viz*. (i) after dilution with the extender, (ii) after equilibration(at 4-5°C for 4 h) and (iii) after 24 h of cryopreservation. Seminal attributes *viz*. progressive motility, sperm viability and membrane integrity (hypoosmotic swelling test [HOST]) were evaluated at all three stages.

### Preparation of BHT

275.44 mg of BHT, Kosher grade (Sigma-Aldrich, St. Louis, USA) was dissolved in 25 ml of ethanol making a BHT concentration of 0.05 M. This solution was then added @ of 0.05 and 0.1ml in the two glass test tubes. Ethanol containing BHT was then evaporated at 37°C in an incubator resulting in sticking of BHT in the inner wall of the test tubes. The semen was extended with GEYT extender up to 80 million spermatozoa/ml. To each of this test tube 5 ml of extended semen was added making a concentration of 0.5 mM BHT (T1) and 1.0 mM BHT (T2). The extended semen along with BHT was then kept at 37°C for 5 min to allow uptake of BHT by spermatozoa. Simultaneously, the same amount of extended semen was added to another test tube (without BHT), which was considered as control group. All the extended semen (with and without BHT) were then filled in 0.25 ml, French straw with the help of filling and sealing machine, equilibrated in a cooling cabinet (temperature 4-5°C) in a straw rack for 4 h.

### Processing of semen for freezing

The extended semen with BHT concentration of 0 mM, 0.5 mM and 1.0 mM were considered as control, treatment group T1 and T2 respectively and were taken for cryopreservation using a biological freezer (liquid nitrogen vapor freezing). These samples containing the BHT were processed for cryopreservation as per the standard protocol (lowering of temperature from 4°C to −10°C @ 5°C/min, −10°C to −100°C @ 40°C/min, −100 to −140°C @ 20°C/min) developed in the semen biology laboratory, DUVASU, Mathura.

### Post-thaw evaluation of sperms

After 24 h of cryopreservation, the straws containing the semen were thawed in thawing unit (IMV, France) maintained at 37°C with holding time kept as 45 s. After thawing the straws were processed for retrieval of the sperms which was followed by physico-morphological evaluation *viz*. progressive motility, sperm viability and sperm membrane integrity (HOST) in the control and test samples (T1 and T2) [12]. Progressive motility was observed on a thermostatically regulated stage, whereas, the sperm membrane integrity was evaluated by exposing the sperms to hypo-osmotic solution (150 mOsmol/L). Sperm viability was enumerated by Eosin-Nigrosin staining [13].

### Statistical analysis

Statistical analyses were performed using Statistical Package for Social Science (SPSS^®^ Version 22.0 for Windows^®^, SPSS Inc., Chicago, USA). Data are presented as mean and their standard error (Mean±standard error of mean). Effect of different inclusion levels of BHT (antioxidant) were analyzed using one-way analysis of variance and significance was tested at 5% level (p<0.05). Duncan’s multiple range test was used to compare the treatment means for various sperm attributes.

## Results

Sperm viability was evaluated in the control sample and in two treatment groups (T1 and T2) during three stages of sperm processing. Sperm viability exhibited significant (p<0.05) difference between control and two treatment groups. Group T2 exhibited the best results in terms of sperm viability (Table-1). A similar trend was seen for sperm motility and membrane integrity as shown in Tables-2 and 3. Progressive motility was significantly (p<0.05) increased in both T1 and T2 group as compared to the control and T2 exhibited the best results in terms of progressive motility in all the three stages of processing. Sperm membrane integrity was significantly (p<0.05) increased in T2 group as compared to both control and T1 group at all three stages of semen processing (Table-3).

**Table-1:** Live spermatozoa in the semen of Hariana bulls extended in GEYT with BHT supplementation during different stages (after dilution and prefreezing) of cryopreservation and following thawing (mean±SE, n=24).

Mean percentage of live spermatozoa			

Stages	Control	Treatment 1	Treatment 2
After dilution	80.59^a^±0.56	83.89^b^±0.54	88.10^c^±0.64
Pre freezing	73.93^a^±0.71	78.42^b^±0.64	82.44^c^±0.67
Post thaw	59.95^a^±0.70	65.39^b^±0.63	70.72^c^±0.78

Means with different superscript letters (a, b, c) differ significantly within a row. Treatment 1=0.5 mM BHT, treatment 2=1.0 mM BHT, significance level=5%. BHT=Butylated hydroxytoluene, GEYT=Glycerolated egg yolk tris, SE=Standard error

**Table 2:** Progressively motile spermatozoa in the semen of Hariana bulls extended in GEYT with BHT supplementation during different stages (after dilution and prefreezing) of cryopreservation and following thawing (mean±SEM, n=24).

Mean percentage of progressively motile spermatozoa			

Stages	Control	Treatment 1	Treatment 2
After dilution	69.46^a^±0.63	73.08^b^±0.59	77.50^c^±0.70
Pre freezing	63.54^a^±0.60	67.75^b^±0.55	71.92^c^±0.65
Post thaw	49.17^a^±0.57	54.50^b^±0.50	59.13^c^±0.60

Means with different superscript letters (a, b, c) differ significantly within a row. Treatment 1=0.5 mM BHT, treatment 2=1.0 mM BHT, significance level=5%. BHT=Butylated hydroxytoluene, GEYT=Glycerolated egg yolk tris, SEM=Standard error of mean

**Table-3:** HOS reactive spermatozoa in the semen of Hariana bulls extended in GEYT with BHT supplementation during different stages (after dilution and prefreezing) of cryopreservation and following thawing (mean±SEM, n=24).

Mean percentage of HOS reactive spermatozoa			

Stages	Control	Treatment 1	Treatment 2
After dilution	78.11^a^±0.58	81.05^b^±0.59	84.92^c^±0.92
Pre freezing	72.46^a^±0.97	75.90^b^±0.67	79.51^c^±0.69
Post thaw	57.34^a^±0.74	62.94^b^±0.64	67.92^c^±0.73

Means with different superscript letters (a, b, c) differ significantly within a row. Treatment 1=0.5 mM BHT, treatment 2=1.0 mM BHT, significance level=5%. BHT=Butylated hydroxytoluene, GEYT=Glycerolated egg yolk tris, SEM=Standard error of mean, HOS=Hypoosmotic swelling

## Discussion

The present study was an attempt to evaluate the beneficial roles of BHT as an antioxidant additive to the semen samples collected from Hariana bulls during three stages of semen processing *viz*. extended semen, semen after equilibration and thawing after 24 h of cryopreservation. The results obtained from the study have been presented in Tables 1-3. Retention of sperm viability is the prerequisite of semen cryopreservation so as to use the sperms for artificial insemination. The cryo damaging effect during cryopreservation induces subtle changes in the sperms which not only causes a reduction in the sperm viability but also reduce motility of the sperms [1]. The major setback to the sperm is provided by the free radicals, which are generated during the time of semen cryopreservation. Free radicals are molecular oxygen exhibiting high reactivity, targeting the sperm plasma membrane, the lipids therein and the sperm DNA. The consequence to these events, there is the induction of cell death by the process of apoptosis leading to an ultimate reduction in the viability of the sperms [14].

Viability of the sperms after the process of freeze-thaw is always a challenging step as 30 to 40% of the reduction in the viability of the sperm is seen. The loss in the sperm viability has been associated with the changes in the temperature, and ultra-low temperature maintained during cryopreservation [1]. The viability of the sperm is also reduced due to the generation of free radicals during the time of cryopreservation leading to lipid peroxidation and ultimate reduction in the sperm viability [4]. To restore the viability, the semen extenders must have enough antioxidant defenses so as to fight against the generated free radicals to minimize the free radical mediated lipid peroxidation [6]. In the present study, the viability of the sperms has been significantly increased in the semen samples containing the BHT as additive. Both the semen samples exhibited an increase in the viability of the sperms as compared to the control samples at all the three stages of sperm evaluation. The addition of BHT has been shown to increase the antioxidant defense and as a consequence to this, there was a resultant increase in the viability % of the sperms [9,10,15,16].

Free radicals generated at homeostatic levels are significant in regulating the sperm physiological functions like sperm capacitation, acrosome reaction, hyperactivation and sperm-oocyte fusion [17]. However, not all reactive oxygen species (ROS) are beneficial to the sperms along with higher levels of free radicals, which induce oxidative stress to the sperms. As a consequence to this, there is substantial damage to the sperms involving the plasma membrane damage, leakage, and increased permeability leading to reduced sperm motility, metabolic activity, longevity and viability [18].

Semen processing during the time of cryopreservation results in dilution of sperm antioxidant system and along with this, the generated free radicals brings damage to the polyunsaturated fatty acids present on the sperm cell membrane [19]. The addition of BHT has a protective role on the sperms as it is postulated that BHT penetrates the sperm cell membrane and thereby exerts its antioxidant defense [20]. BHT penetration also increases the membrane fluidity and flexibility of the sperms along with conversion of peroxyl radicals to hydroperoxides. By these mechanisms, BHT acts as an antioxidant to the cryopreserved sperms. Studies also have shown the role of BHT as an anti-lipid peroxidation agent and hence offers protection to the sperms during cryopreservation [21].

Studies in many species of domestic animals have also been shown that BHT exerts a protective effect on the sperms during freeze-thaw process. The beneficial effects of BHT are also dependent on the concentration in the extender, which is also species dependent and specific. Earlier studies have reported the beneficial role of BHT is dependent on BHT concentration, sperm membrane composition, time for incubation and the type of extender used for the process of cryopreservation [19].

Extension of the semen with desired low concentration of BHT is the prerequisite to get the best results in terms of sperm protection. Bull semen extended with 0.5-1 mM BHT has been shown better results as compared to 3 mM or more. Supplementation with higher concentrations of BHT has shown detrimental effects on the sperms opted for cryopreservation. In our study, we reported the similar concentration of BHT offering a protective effect on the sperm after freezing and thawing. BHT concentration has also exhibited a species specific variation [19-21].

Progressive motility serves as the index of fertilization and serves as the most significant determinant of the sperm capability for bringing out fertilization. Motility is a complex factor, which is regulated strictly by the flagellar assembly proteins along with the generated ATP by the mitochondria [5]. Sperm motility has been shown to reduce by 50% after freezing and thawing in most of the species of the animal, and this may be a probable factor behind the reduction in the sperm quality [1]. That is why it is imperative to keep the sperm motility at a higher level after cryopreservation so as to achieve optimal fertility. The loss in the motility of the sperms has also been associated with the increase in free radicals during cryopreservation and lipid peroxidation [6]. To restore the motility and to reduce the free radical mediated oxidative stress, it is essential to improve the antioxidant defense to the sperms opted for cryopreservation. This optimization can be achieved by supplementing the extender with antioxidant additives [22]. In the present study, we noted a significant increase in the post-thaw motility in the samples containing the BHT as an additive. After dilution along with equilibration, the sperms exhibited a higher motility as compared to the control non-supplemented groups. The study defined the antioxidant role of BHT in the improvement of the sperm motility.

Sperm membrane integrity is the index of sperm function as well as an indication of metabolic status. Membrane integrity is associated with keeping the sperms active in terms of regulation of the osmotic balance of the cells. The loss in the membrane permeability is a common limitation in the sperms opted for freezing and thawing [7]. Membrane leakiness is due to the peroxidative damage caused by the free radicals as a result to the dilution of antioxidant defense. The membrane intactness can be retained by increasing the antioxidant defense and thereby reducing the free radical mediated oxidative damage to the cells [6]. BHT supplementation has been shown the beneficial effect to the sperms at all the three stages of handling and processing of sperms.

For all the parameters consider under this study, BHT used to a level of 1.0 mM BHT (T2) did not have any toxic effect to the spermatozoa. Hence, our result indicates that 1.0 mM BHT is suitable for freezing bull spermatozoa in GEYT extender having 20% egg yolk and 7% glycerol with 80 millions/ml sperm concentration of extended semen.

Extension of the semen with two concentrations of BHT improved the post-thaw motility, viability and membrane integrity of Hariana bull sperms. The exact mechanism behind the process of improvement of sperm quality after the addition of BHT has not been understood from the study, but it was probably due to the action of BHT as an antioxidant and a consequence reduction in the lipid peroxidation [20,23]. Polyunsaturated fatty acid content of sperm plasma membrane is higher and is highly prone to lipid peroxidation during the process of cold/heat shock in freezing and thawing. BHT penetrates the plasma membrane of the sperm cells and prevents the cell membrane from free radicals [11]. That is why BHT acts as an exogenous antioxidant. It is also possible that BHT in the added samples may have boosted the antioxidant defense to the sperms.

The present study investigated whether the addition of BHT has improved the quality of Hariana bull sperm after dilution, equilibration, and thawing. The results revealed a significant improvement in the quality of sperm in all the three stages with the addition of 1 mM BHT. The characters evaluated were progressive motility, sperm viability and membrane integrity. The findings of the study revealed a significant improvement in the sperm quality with the addition of both the concentrations of 0.5 mM and 1 mM BHT.

## Conclusion

Extender containing the exogenous BHT revealed better post-thaw semen quality as compared to the extender without BHT. Addition of BHT at a concentration of 1mM revealed better results as compared to 0.5 mM. BHT augmented post-thaw semen quality by the reduction in the generation of free radicals and thereby a consequent reduction in lipid peroxidation. Further studies are required to understand the role of BHT in improving the sperm quality and whether, this addition translates into the improved fertility of artificially inseminated sperms.

## Authors’ Contributions

AP was the MVSc. Scholar of the department who carried out experimental research work and laboratory analysis of data. AS was guide of AP, under whose supervision the thesis was submitted. AS planned and designed the experiment. AK helped in design of experiment. DKS help in manuscript preparation. DY, SSY and AK (MVSc. Scholars) helped in sterilization of equipment used in semen experiment, preparation of semen extender/stains for semen evaluation and processing of semen during the research work. All authors read and approved the final manuscript.
